# Accuracy of commercial intraoral scanners

**DOI:** 10.1117/1.JMI.8.3.035501

**Published:** 2021-05-24

**Authors:** Mattia Sacher, Georg Schulz, Hans Deyhle, Kurt Jäger, Bert Müller

**Affiliations:** a University of Basel, Biomaterials Science Center, Department of Biomedical Engineering, Faculty of Medicine, Allschwil, Switzerland; b Praxis-Team St. Margarethen Binningen, Binningen, Switzerland; c Diamond Light Source, Oxfordshire, United Kingdom; d Argodentis Zahnmedizin, Aarburg, Switzerland

**Keywords:** three-dimensional accuracy evaluation, micro computed tomography, stereolithography printer, full-arch scanning, registration, deviation field

## Abstract

**Purpose:** In dental offices, there is a trend replacing conventional silicone impressions and plaster cast models by imaging data of intraoral scanners to map the denture and surrounding tissues. The aim of the study is the analysis of the accuracy of selected commercially available scanners. The accuracy is considered as the main drawback in comparison to the conventional approach.

**Approach:** We evaluated the reproduction performance of five optical scanners by a direct comparison with high-resolution hard x-ray computed tomography data, all obtained from a polyetheretherketone model with similarity to a full-arch upper jaw.

**Results:** Using the software GOM Inspect (GOM GmbH, Braunschweig, Germany), we could classify the intraoral scanners into two groups. The more accurate instruments gave rise to the following precision values: 35  μm (TRIOS^®^ 3, 3shape, Copenhagen, Denmark), 43  μm (CS 3600, Carestream, Atlanta, Georgia), and 46  μm (3M™ True Definition Scanner, 3M ESPE, St. Paul, Minnesota). The less precise systems yielded 93  μm (Medit i500, Medit corp., Seongbuk-gu, South Korea) and 97  μm (Emerald™, Planmeca Oy, Helsinki, Finland).

**Conclusions:** The selected scanners are suitable for single crowns, small bridges, and separate quadrants prostheses. Scanners based on triangulation are hardly appropriate for full-arch prostheses. Besides precision, however, the choice of the scanner depends on scanning time, intraoral-camera size, and the user’s learning curve. The developed protocol, which includes three-dimensional (3D) imaging and advanced computational tools for the registration with the design data, will be increasingly used in geometrical metrology by nondestructive procedures to perform dimensional measurements with micrometer precision and is capable for detailed 3D geometrical models reconstruction.

## 1 Introduction

An increasing number of dental treatments require the highly precise impression of the oral situation, and the quality of the treatment and the related success of the therapy depend on the correctly performed impression. For any fixed and removable prosthetics, the impression is fundamental, while for less critical cases, irreversible hydrocolloid materials, such as alginate, are used in conventional workflow. With an accuracy below 150  μm, these hydrocolloidal impressions are usually less precise than digital impressions;^1^ however, conventional impressions, taken with rigid trays and elastomeric materials, are so accurate that they are considered as the gold standard.^2^ Conventional impressions require the production of a plaster cast model.

In the digital workflow, no cast is needed, but depending on the procedure, a physical model with the necessary accuracy requirements has to be produced. These models are mostly three-dimensionally printed using stereolithography apparatuses (SLA), and they feature clinically acceptable accuracy.^3^ Intraoral scanners (IOS) are gaining importance in dental offices, and so they are therefore responsible for a paradigm shift in prosthetic dentistry.^2^^,^^4^ The latest generation of video-based systems (IOS) seems to be more accurate, faster, and more efficient in clinical application than previously employed devices, and they are even suitable for less experienced practitioners, because of their simplified handling.^1^^,^^5^^,^^6^ IOSs offer advantages compared to conventional impressions, and digital impressions are time-efficient and much more comfortable for patients. In particular, patients suffering from the gag reflex benefit.^7^

IOSs use suitable cameras for the optical detection of the denture that collect point clouds on a path proposed by the suppliers and determined by the dentist. The physical principles of IOSs can be divided into confocal techniques with structured light with wavelengths ranging from ultraviolet to red color and the triangulation of projected pattern using preselected colors.^8^^–^^10^ The description of the obtained surface in the three-dimensional (3D) space happens in the standard triangulation language (STL)-format.

Crowns and bridges can be directly manufactured using computer-assisted design (CAD)/CAM, or, alternatively, models can be produced by means of stereolithography.^11^^,^^12^ The main disadvantages of IOS are acquisition costs and intricate access to light-tight areas, including subgingival preparations.

The technology involved in intraoral scanning has significantly improved since 1980, when the first CEREC was introduced to the market.^13^ In the meantime, intraoral scanning has been established in a wide range of indications.^14^ Intraoral scans are used in prosthodontics for inlays/onlays, crowns, frameworks, fixed and removable partial prostheses, posts and cores, crowns and bridges and for digital smile design. In orthodontics, digital impressions serve as the basis for treatment planning, for custom-made devices and (transparent) aligners.^15^ In implant surgery, intraoral scans are integrated into the digital workflow to plan clinical cases and produce surgical guides,^16^ and they are increasingly considered more often as highly accurate, exhibiting no difference in comparison to conventional impressions for crowns and fixed dental prostheses of limited length.^17^^,^^18^ In the current literature, however, long-spanning fixed restorations or totally removable prostheses, which include six or more elements, are deemed somewhat problematic.^1^^,^^15^ The available studies about the precision of IOS hardly cover the latest generation of devices. The progress in technology by comprising high-resolution cameras for data acquisition and high-performance software for the polygonal mesh generation of the model has led to sudden improvement of the IOS performance.^19^ The advancement of software particularly has enabled solutions not otherwise possible with conventional impression technology. Here, it is possible not only to analyze imaging data in detail but also to modify crown preparations during treatment. Potential deficiency in the obtained impressions can be minimized or even fully removed, and related shade measurements assist in the determination of crown color. To educate the patient, the captured images can be presented immediately after scanning, while a series of scans from the same patient can be the basis of monitoring changes over time.

There is an increasing number of comparative studies addressing accuracy, which refers to both trueness and precision.^8^^–^^10^^,^^20^ Advanced micro computed tomography systems provide an accuracy of <10  μm for objects as large as a human jaw.^2^ Therefore, this approach is also applied for this study.^21^ The study should quantitatively answer the question how far the five selected IOS systems reach the clinically required accuracy of 120  μm.^18^ Employing this example, we will discover the assets and drawbacks of registering the nondestructively acquired 3D data of microtomography and optical scanning with the design for metrology of 3-in. solids down to a very few micrometers.

## 2 Methodology

### 2.1 Model Fabrication

**Fig. 1:**
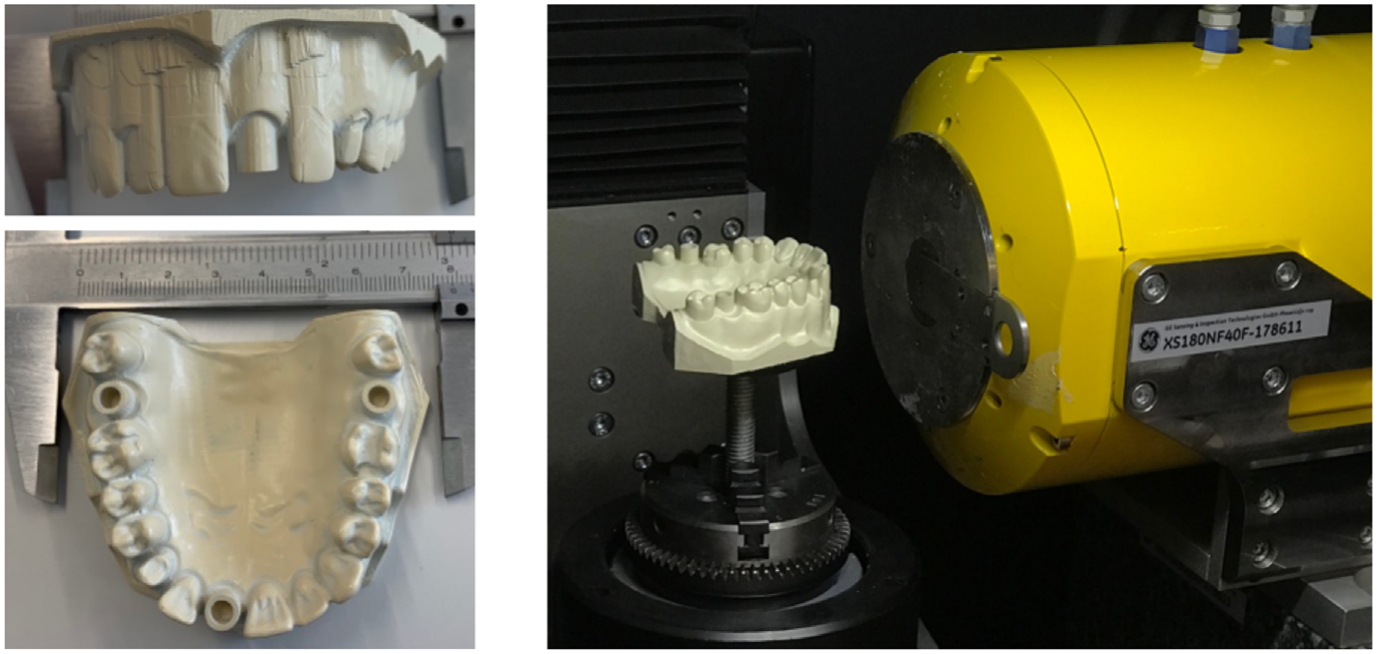
The PEEK model’s size corresponds to that found in the human body, as displayed by the photographs on the left. Three well-defined hollow cylinders were incorporated to determine the precision of the IOSs. The other photograph shows the placement of the model on the rotation stage in the CT-system nanotom^®^ m (phoenix|x-ray, GE Sensing & Inspection Technologies GmbH, Wunstorf, Germany).

The anatomic model of a maxillary full denture, based on standard working models (frasaco GmbH, Tettnang, Germany), was created with CAD and commercially available software (Meshmixer, Autodesk Inc., San Rafael, California). The parallel cylinders, with a predefined inner diameter and a depth of 4 mm, were placed at tooth positions 17 (C1), 21 (C2), and 27 (C3) as reference elements (see photograph in Fig. 1). A characteristic crown preparation at tooth 23, and an inlay preparation at tooth 16, simulated a normal prosthetic situation. The master model was milled out of an industrially manufactured polyetheretherketone (PEEK) block (Denseo PEEK blank, Denseo GmbH, Aschaffenburg, Germany) on a five-axis computerized-numerical-control milling machine (SilaMill 5, vhf camfacture AG, Ammerbuch, Germany). PEEK is a high-performance polymer, and it is known to be dimensionally stable ^22^ and is also used in medical applications, see, e.g., Ref. 23. An extension on the back of the model served for mounting purposes.

In addition, two other models were fabricated using the same design. A photopolymer resin, namely dental model resin (Formlabs, Somerville, Massachusetts), was used within the SLA printers form 2 (Formlabs, Somerville, Massachusetts) and with a layer thickness of 25  μm. Models fabricated by the stereolithography technology were washed with isopropyl alcohol for a period of 15 min. Subsequently, they were postcured with UV light under an inert gas atmosphere. The density of the models was experimentally determined to $1.59\;g/{\mathrm{cm}}^{3}$, which is significantly higher than the value of the raw material at $1.12\;g/{\mathrm{cm}}^{3}$. This result is an indicator of cross-linking at the 405-nm wavelength.

### 2.2 Reference Generation

The high-resolution CT (μCT) scans of the master model, described already, served as the reference data. Tomographic data were recorded using the advanced conventional system nanotom^®^ m (phoenix|x-ray, GE Sensing & Inspection Technologies GmbH, Wunstorf, Germany), as shown in Fig. 1. This system is equipped with a nanofocus tube with a maximal acceleration voltage of 180 kV, which produces power of up to 15 W. For data acquisition, we employed the maximal acceleration voltage and a beam current of 30  μA. To shift the mean photon energy to higher values, a 0.5-mm-thin copper film was placed behind the transmission target. We recorded 1600 radiographs throughout 360 deg. The exposure time for the first two datasets was set to 3 s per radiograph, whereas it was increased to 9 s for the third and to 24 s for the fourth dataset. To investigate the repeatability of the μCT-system and the impact of the cone beam, the following source–sample distances (SSDs) and source–detector distances (SDDs) were positioned. For the first and the second scans, SSD and SDD were 78.75 and 225.00 mm, respectively. For the third scan, we used SSD 157.50 mm and SDD 450.00 mm, and for the fourth scan SSD 210.00 mm and SDD 600.00 mm were used. Exposure times were adapted to maintain comparable intensities, and the effective pixel length corresponded to 35  μm. These approaches keep the magnification constant. A large SSD limits the angle of the cone beam and might improve the accuracy but leads to significantly longer exposure times.

In a previous study,^2^ we have captured a dental model using the nanotom^®^ m and the tactile coordinate measuring machine Leitz PMM 864 (Hexagon Metrology GmbH, Wetzlar, Germany). Both approaches provided true micrometer resolution. Consequently, the micrometer precision of the nanotom^®^ m system for centimeter distances was validated.

For visualization purposes, the acquired volume data were processed by means of VGStudio MAX (Volume Graphics, Heidelberg, Germany). In addition, the data size was reduced and converted into the STL format. This conversion enabled us to compare the tomography data with data for the intraoral scans.

### 2.3 Data Acquisition, Using Intraoral Scanners

This study comprises five IOS systems, namely the 3M™ True Definition Scanner (3M ESPE, St. Paul, Minnesota), the TRIOS^®^ 3 (3shape, Copenhagen, Denmark), the CS 3600 (Carestream, Atlanta, Georgia), the Medit i500 (Medit corp., Seongbuk-gu, South Korea), and the Emerald™ (Planmeca Oy, Helsinki, Finland). With each system, the master model was scanned 10 times to obtain the necessary statistics. For this purpose, the model was mounted in the anatomically correct upright position. One trained examiner (M.S.) performed all of the scans in identical conditions (light, temperature, etc.), and scanner handling, the use of powder, and the scan path were carried out according to the manufacturer’s guidelines. 3M recommended the use of powder to improve the scanning results. To prepare the model as recommended, a thin layer of powder (3M Powder Sprayer; 3M, St. Paul) was applied to the model. The scans with the other four systems were taken without the application of powder, i.e., in a powder-free fashion.

### 2.4 Data Procession and Evaluation

**Fig. 2:**
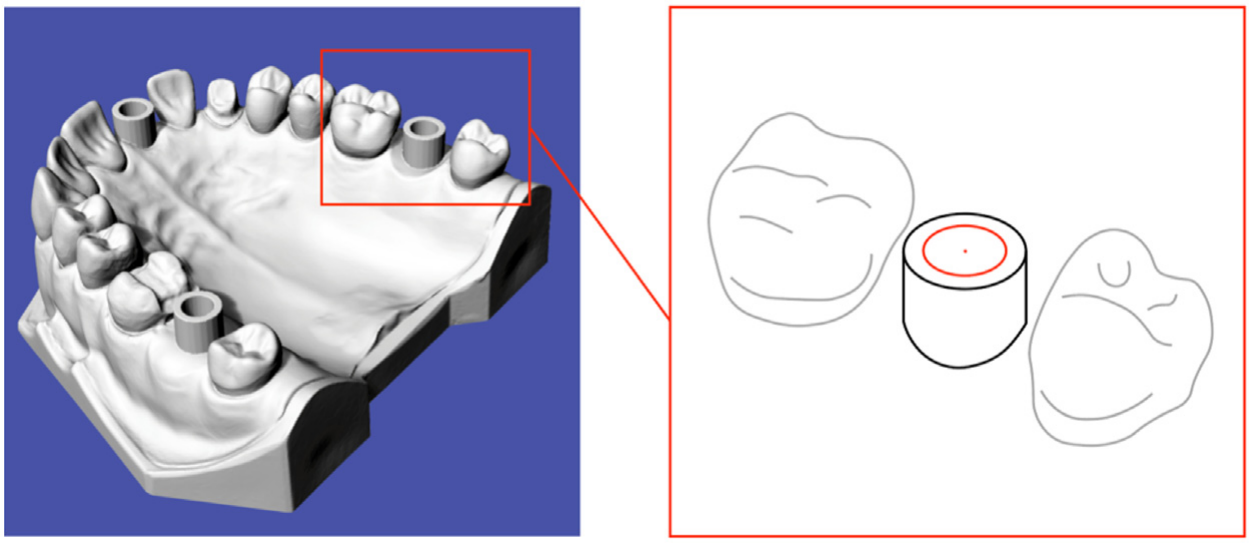
Scheme for determining the center points of the hollow cylinders via the Gaussian best-fit method.

**Fig. 3:**
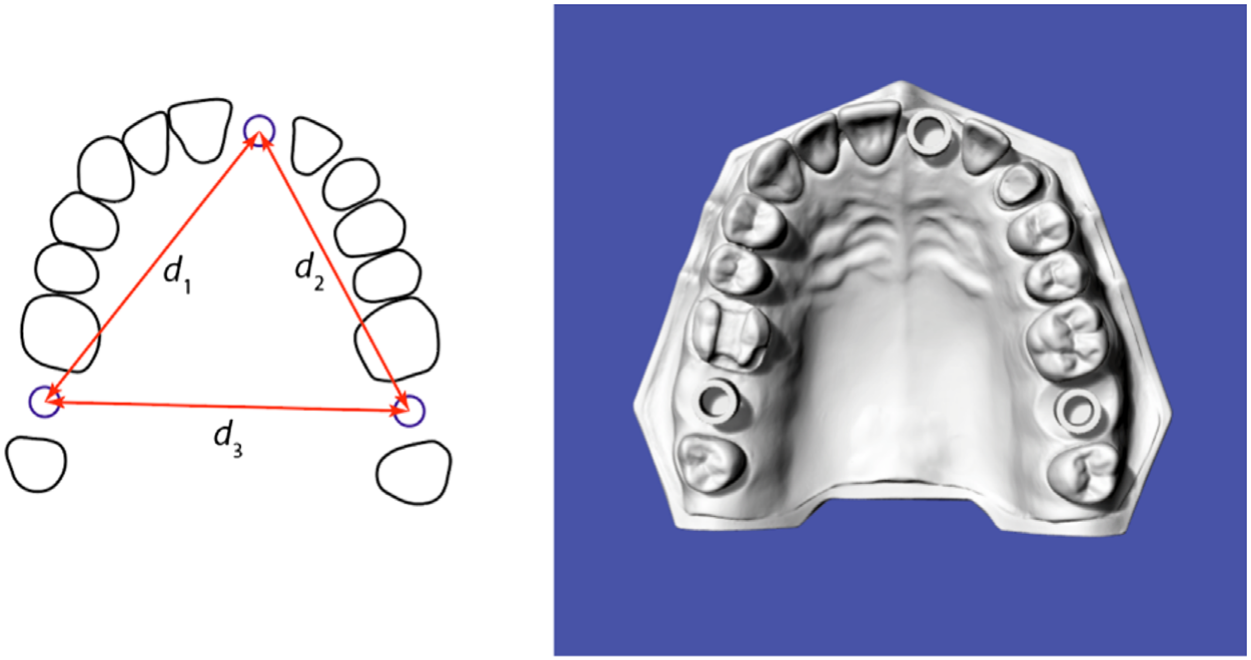
Scheme for specifying the three distances. Distance $d_{1}$ characterizes the length between 17 and 21, distance $d_{2}$ is the length between 21 and 27, and distance $d_{3}$ corresponds to the length between 17 and 27.

The measurements and analyses were performed using the well-established software GOM Inspect (GOM GmbH, Braunschweig, Germany). The positions of the three hollow cylinders were determined using their center points, identified via the Gaussian best-fit method, see Fig. 2. The three distances were derived from tomography and IOS data individually, see Fig. 3.

To compare accuracy and precision, these data were matched to the reference. The global best-fit deviation was calculated for each scan, in order to visualize the displacement field, see what follows.

## 3 Results and Discussion

### 3.1 Measurements with nanotom^®^ m

To confirm the well-known stability of the μCT-system and the reproducibility of the tomographic data acquisition, the first two scans were performed in identical conditions. As expected, differences were within the micrometer range, and even for distances between 4 and 5 cm, as given by the spacing between the hollow cylinders, the differences were well below the voxel length of 35  μm. For scan 1, we found $d_{1}=45.444\;\mathrm{mm}$, $d_{2}=41.622\;\mathrm{mm}$, and $d_{3}=48.098\;\mathrm{mm}$. For scan 2, the selected distances were determined to $d_{1}=45.450\;\mathrm{mm}$, $d_{2}=41.627\;\mathrm{mm}$, and $d_{3}=48.101\;\mathrm{mm}$. This result, i.e., differences of 4 to 6  μm or relative deviations by about $10^{-4}$, indicates that imaging modality and the method for determining longitude are appropriate choices for precision measurements. As a consequence, we assumed an error bar of 6  μm for establishing the spacing between the hollow cylinders.

To evaluate the impact of the cone-beam geometry in the nanotom^®^ m on the accuracy, two scans with modified sample positions with respect to source and detector were performed. The experimentally determined distances between the hollow cylinders of scan 3 were $d_{1}=45.468\;\mathrm{mm}$, $d_{2}=41.640\;\mathrm{mm}$, and $d_{3}=48.127\;\mathrm{mm}$. The values for scan 4 amounted to $d_{1}=45.480\;\mathrm{mm}$, $d_{2}=41.647\;\mathrm{mm}$, and $d_{3}=48.134\;\mathrm{mm}$. Compared to the set value, given by the STL file with $d_{1}=45.503\;\mathrm{mm}$, $d_{2}=41.676\;\mathrm{mm}$, and $d_{3}=48.192\;\mathrm{mm}$, we identified values only 23, 29, and 58  μm smaller. Although the discrepancies amount only to 1/10th of a percent and less than two pixel lengths, the phenomenon was still detectable. Therefore, tomographic data acquisition for the three-dimensionally printed models was performed with the parameters in scan 4. It should be noted, however, that the potential gain in accuracy meant that data acquisition took seven times longer to complete than the standard approach.

### 3.2 Geometry of the PEEK Model

The PEEK model was generated from the STL-data by a milling machine, it is therefore unknown how far the model corresponds to the desired geometry. Comparing the STL data with the tomography data, one obtains a superposition of deficiencies from milling and imaging. Previous studies with the nanotom^®^ m, however, demonstrate true micrometer accuracy, which is further supported by the measurements described above. Therefore, we can reasonably estimate the accuracy of the PEEK machining through a direct comparison of STL-data with high-resolution tomography data.

**Fig. 4:**
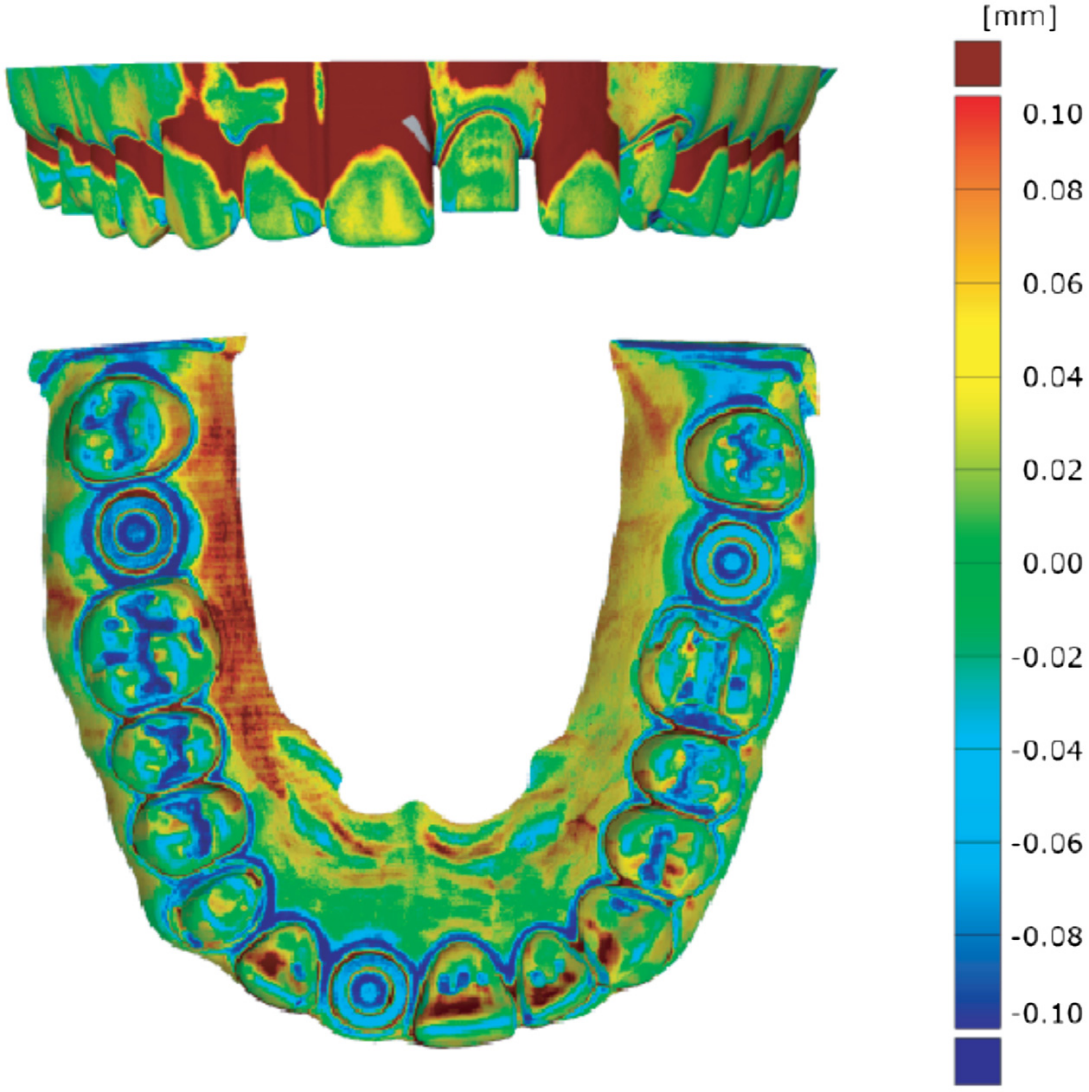
3D representation of the differences between the desired geometry, given by the STL-file and the data from scan 4 and according to the color bar and the related values on the right. Perfect agreement is represented by the green color. Obviously, the milling tool provided a reasonable result, although in some areas more material than desired was removed (blue color), while other areas show excess material (red color). One can further observe that the hollow cylinders are larger in size than planned, but because we only consider the center points, the determination of distances is hardly affected.

Figure 4 elucidates the differences between the design and the measured data using the color code on the color bar. The red color indicates positions, where material more than 100  μm thick should be further removed. The blue color represents positions, where the milling tool removed more than originally desired, and the green color shows positions in perfect agreement. Since the hollow cylinders are almost identical, their axes and related center points are not affected. Measurement of the distances described already is therefore meaningful.

### 3.3 Geometry of the SLA Models

In specific situations, the digital workflow has to be modified, which might require a physical model to be generated from the IOS data. Here, the method of choice is stereolithography,^24^ but current clinical experience shows that these SLA-models are less accurate than conventional impressions. To quantify this level of accuracy, we produced two SLA-models and used tomographic imaging and registration.

**Fig. 5:**
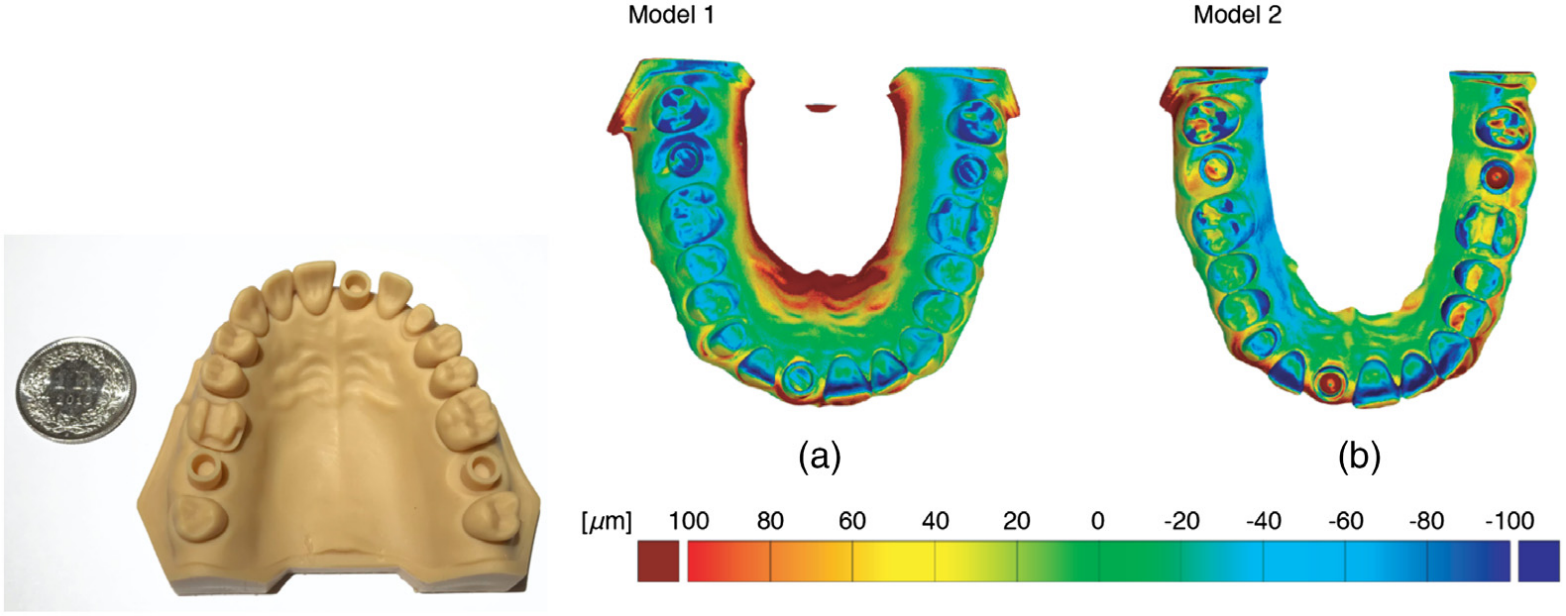
3D representation of the differences between the desired geometry, given by the STL-file, we used for the model preparation (photograph on the left) and the high-resolution tomography data of the two 3D-printed models. The green color shows parts without differences, whereas the red and blue colors indicate excess and deficiency of material, respectively.

Based on the high-resolution tomography measurements performed as described already, the distances $d_{1}$, $d_{2}$, and $d_{3}$ were extracted. For the first SLA-model, the three distances corresponded to 45.611, 41.830, and 48.210 mm, whereas for the second SLA model, we found 45.753, 41.831, and 48.247 mm, meaning that the two models were not exactly the same in terms of geometry. The differences were in the submillimeter range, see Fig. 5. Comparison with STL-data, however, is the benchmark. The average deviation along the full arch amounted to (49±9)  μm. For the distances considered within this study, the deviations accounted for $\Delta d_{1}=108\;\mu m$, $\Delta d_{2}=154\;\mu m$, and $\Delta d_{3}=18\;\mu m$ (first SLA model), and $\Delta d_{1}=250\;\mu m$, $\Delta d_{2}=155\;\mu m$, and $\Delta d_{3}=55\;\mu m$ (second SLA model). These differences between the desired design and the actual physical models clearly indicate the limitations of the stereolithography printers currently used in dental offices.

### 3.4 Accuracy of the Intraoral Scanners

The results obtained from the 10 independent experiments with each of the IOS systems are listed in Table 1. These numbers show the reproducibility of the individual devices, when an experienced dentist has performed data acquisition according to the guidelines provided by suppliers. The data scattered within a few tens of micrometers.

**Table 1:** Measured distances $d_{1}$, $d_{2}$, and $d_{3}$ (in mm) obtained from the IOS systems used. The data determined from the μCT scans ($d_{1}=45.480\;\mathrm{mm}$, $d_{2}=41.647\;\mathrm{mm}$, and $d_{3}=48.134\;\mathrm{mm}$) can be regarded as ground truth.

3M TDS			TRIOS^®^ 3			CS 3600			Medit i500			EMERALD™		
$d_{1}$	$d_{2}$	$d_{3}$	$d_{1}$	$d_{2}$	$d_{3}$	$d_{1}$	$d_{2}$	$d_{3}$	$d_{1}$	$d_{2}$	$d_{3}$	$d_{1}$	$d_{2}$	$d_{3}$
45.577	41.695	48.118	45.536	41.749	48.256	45.567	41.703	48.128	45.458	41.607	48.036	45.439	41.665	48.144
45.545	41.708	48.111	45.548	41.728	48.106	45.519	41.698	48.027	45.440	41.627	48.120	45.499	41.512	48.349
45.534	41.711	48.094	45.568	41.721	48.244	45.564	41.707	48.185	45.456	41.573	47.907	45.411	41.528	48.192
45.535	41.693	48.035	45.53	41.729	48.279	45.536	41.658	48.147	45.391	41.568	47.857	45.499	41.537	47.891
45.559	41.707	48.001	45.563	41.723	48.309	45.568	41.673	48.202	45.405	41.567	47.705	45.479	41.594	47.887
45.567	41.726	48.122	45.538	41.723	48.287	45.550	41.676	48.157	45.380	41.562	47.62	45.484	41.527	47.874
45.574	41.719	48.190	45.514	41.715	48.088	45.593	41.679	48.194	45.357	41.55	47.595	45.490	41.575	48.472
45.568	41.709	48.188	45.524	41.745	48.274	45.572	41.677	48.219	45.349	41.576	47.499	45.578	41.506	48.424
45.573	41.718	48.160	45.519	41.705	48.338	45.580	41.686	48.243	45.316	41.502	47.449	45.552	41.597	48.561
45.568	41.705	48.122	45.534	41.756	48.403	45.559	41.725	48.235	45.317	41.576	47.430	45.510	41.628	48.645

To determine the accuracy of the IOS systems in measuring the three selected distances, we compared these values with tomography data for the PEEK model. For the 3M™ True Definition Scanner (3M ESPE, St. Paul, Minnesota), which is based on pulsed blue light and active wavefront sampling,^8^ the deviations for $d_{1}$ were (80±14)  μm, for $d_{2}$ (62±8)  μm, and for $d_{3}$ we found −(20±7)  μm. For the TRIOS^®^ 3 (3shape, Copenhagen, Denmark), a structured light scanner using confocal microscopy principle,^9^ the length deviations amounted to (57±18)  μm, (82±16)  μm, and (124±97)  μm, respectively. The CS 3600 (Carestream, Atlanta, Georgia), a system applying four-color structural light,^10^ provided (81±22)  μm, (41±20)  μm, and (40±64)  μm, respectively. Using the Medit i500 (Medit Corp., Seongbuk-gu, South Korea), based on triangulation, we found −(93±54)  μm, −(76±33)  μm, and −(41±247)  μm. Finally, the Emerald™ (Planmeca Oy, Helsinki, Finland), a system applying tree-color projected pattern for triangulation,^10^ yielded $d_{1}=(14\pm 48)\;\mu m$, $d_{2}=-(80\pm 54)\;\mu m$, and $d_{3}=(110\pm 291)\;\mu m$. The error bars corresponded to the standard deviations derived from the 10 measurements. Whereas the data were generally within a tenth of a millimeter, one recognizes some trends. The results of the Medit i500 scanner, for example, gave rise to values below the selected ground truth.

Considering the combination of the three selected distances, one can directly compare the performance in length measurements of the five IOS systems by means of the median values and the related variances. From the lowest to the highest median amplitudes, the Emerald™ gained (2±34)  μm, the CS 3600 achieved (58±2)  μm, the 3M™ True Definition Scanner scored (59±4)  μm, the TRIOS^®^ 3 reached (79±4)  μm, and the Medit i500 led to −(98±45)  μm.

**Fig. 6:**
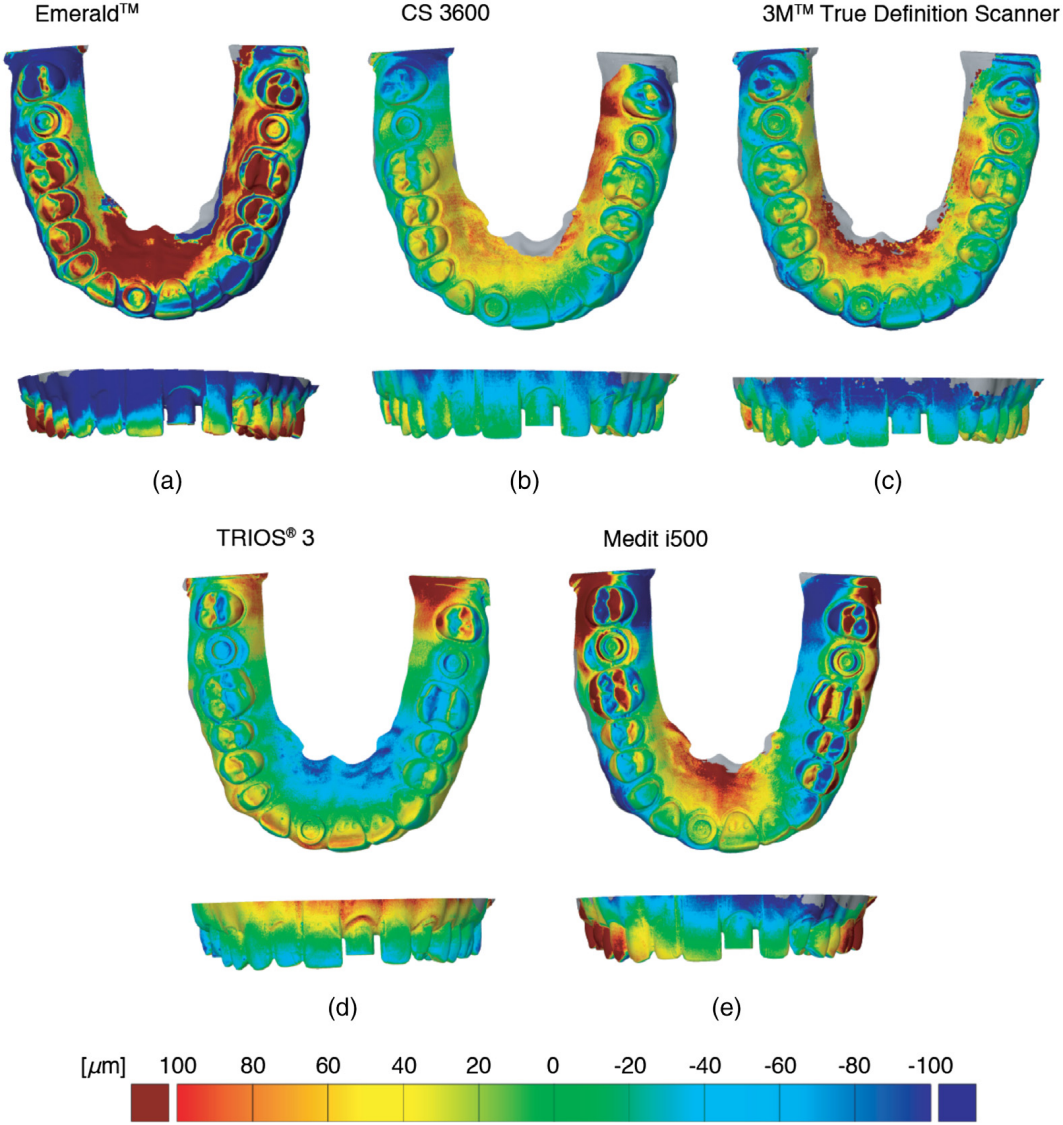
Color-coded deviation field of the IOS derived from the difference to the selected ground truth, i.e., the high-resolution tomography experiments using nanotom^®^ m. The green color shows agreement, whereas the red and blue colors point the locations of positive and negative deviations.

The average values, however, do not represent the full story. Therefore, the color-coded deviation fields are shown in Fig. 6. Although the Emerald™ IOS reproduced the three selected distances perfectly well, the strong color gradients indicate the challenging handling and a relatively weak reproducibility. Consequently, the CS 3600, the 3M™ True Definition Scanner, and the TRIOS^®^ 3 might be the better choice concerning reproducible and accurate measurements within the oral cavity of the patient. The results displayed in Fig. 6 also show that the Medit i500 is less precise than other IOS systems.

Using the software GOM Inspect (GOM GmbH, Braunschweig, Germany), one finds a similar result. This software provided the following precision values: TRIOS^®^ 3—35μm, CS 3600—43  μm, and 3M™ True Definition Scanner—46  μm. The other two systems yielded less precise data: Medit i500—93  μm and Emerald™—97  μm. If only a single quadrant is considered, one finds another picture; therefore, the selection of the best available IOS system depends on the clinical case, the training of the dentist, and the convenience.

### 3.5 Accuracy of Single-Crown Preparation

To analyze the single tooth preparation and the IOS-accuracy on a smaller length scale, Tooth 16 was considered. The mean deviation was determined in the same manner as for the full arch, i.e., n=10. The IOS-data of the single-tooth preparations demonstrated the expected performance of the systems included into the study. The accuracy is almost perfect, because the number of images to be stitched is limited. For the tooth stump (tooth 16), we found the following precision values: TRIOS^®^ 3—(9±5)  μm, CS 3600—(10±5)  μm, Medit i500—(11±1)  μm, and 3M™ True Definition Scanner—(12±1)  μm. Only one system yielded a significantly less precise dataset: Emerald™—(47±9)  μm.

### 3.6 Limitations Related to Clinical Conditions

It should be noted that the results of IOS cannot be fully judged from an *in vitro* study. The dentists have to treat also edentulous patients, to consider the difficulties in controlling the salivary flow, and to account for the presence of restorative and prosthetic works in the dental arch. The usability of the selected IOS device depends the learning curve of the dentist and the size of intraoral-camera size with respect to the space and accessibility of the oral cavity as well as the available and necessary scanning time to record the 3D surface data.

## 4 Conclusions

The accuracy of the nanotom^®^ m data was validated in a previous study.^2^ Replacing the previously used metal cast with the mechanically machined PEEK model, we were able to reduce significantly streak artifacts. Neither full dentition model included edentulous areas, which can be more difficult to scan and could have affected the results.^25^ This experimental study avoided the presence of soft tissues, saliva, blood, filling materials, or space limitations, which often can compromise the accuracy of the scan data in a clinical setting.

Compared to the study presented herein, it is more challenging to determine the precision of IOS systems in an *in vivo* setting. In such studies, the reference model is digitized after a conventional impression and may contain related errors. There are only a few full-arch *in vivo* studies, see, for example, Ref. 1 and an older study;^26^ the results nevertheless correlate with our findings. Physiological tooth mobility can range from 30 to 100  μm,^27^ and the clinically acceptable limit of the marginal gap of a crown is generally 120  μm.^18^

For the full arch, the IOS systems considered herein show reasonable results, although the precision of at least some systems should be improved. In a separate quadrant, however, the devices reach the desired performance.

If the situation requires a physical model, which is then produced using stereolithography technology, full-arch accuracy is often not achieved—contrary to the conventional silicone impressions. As a consequence, they are generally considered as unsuitable for larger prosthetic reconstructions.^2^^,^^3^ In this study, we incorporated only two printers from one model, and thus, the obtained data have limited informative value.

Besides precision, there are further factors including scanning time, the learning curve, or intraoral-camera size, each of which strongly affects the usability of an IOS device. Nevertheless, we can state that current digital impressions exhibit micrometer accuracy and generally produce clinically acceptable data. All analyzed IOS devices are suitable for generating 3D data for working models, single crowns, and small bridges.

Since the accuracy of the digital impressions is fundamental for most clinical applications and the systems differ significantly, it is preferable to use one of the more accurate instruments, which circumvent triangulation, namely the TRIOS^®^ 3, the CS 3600, and the 3M™ True Definition Scanner for the full-arch scanning.

## References

[1] Ender A. Attin T. Mehl A., “*In vivo* precision of conventional and digital methods of obtaining complete-arch dental impressions,” J. Prosthet. Dent. 115(3), 313–320 (2016). JPDEAT 0022-3913 10.1016/j.prosdent.2015.09.011 26548890

[2] Vögtlin C. et al., “Comparing the accuracy of master models based on digital intra-oral scanners with conventional plaster casts,” Phys. Med. 1, 20–26 (2016). 10.1016/j.phmed.2016.04.002

[3] Brown G. B. et al., “Accuracy of 3-dimensional printed dental models reconstructed from digital intraoral impressions,” Am. J. Orthod. Dentofacial Orthop. 154(5), 733–739 (2018). AJOOEB 0889-5406 10.1016/j.ajodo.2018.06.009 30384944

[4] Seelbach P. Brueckel C. Wostmann B., “Accuracy of digital and conventional impression techniques and workflow,” Clin. Oral Investig. 17(7), 1759–1764 (2013). 10.1007/s00784-012-0864-4 23086333

[5] Lim J. H. et al., “Comparison of digital intraoral scanner reproducibility and image trueness considering repetitive experience,” J. Prosthet. Dent. 119(2), 225–232 (2018). JPDEAT 0022-3913 10.1016/j.prosdent.2017.05.002 28689906

[6] Treesh J. C. et al., “Complete-arch accuracy of intraoral scanners,” J. Prosthet. Dent. 120, 382–388 (2018). 10.1016/j.prosdent.2018.01.005 29724554

[7] Mangano A. et al., “Conventional vs digital impressions: acceptability, treatment comfort and stress among young orthodontic patients,” Open Dent. J. 12, 118–124 (2018). 10.2174/1874210601812010118 29492177 PMC5815028

[8] Fukazawa S. Odaira C. Kondo H., “Investigation of accuracy and reproducibility of abutment position by intraoral scanners,” J. Prosthodontic Res. 61(4), 450–459 (2017). 10.1016/j.jpor.2017.01.005 28216020

[r9] Lindström M. J. R. et al., “Volumetric measurement of dentoalveolar defects by means of intraoral 3D scanner and gravimetric model,” Odontology 107(3), 353–359 (2019). 10.1007/s10266-018-00410-6 30617638 PMC6557874

[10] Nagy Z. et al., “Comparing the trueness of seven intraoral scanners and a physical impression on dentate human maxilla by a novel method,” BMC Oral Health 20(1), 97 (2020). 10.1186/s12903-020-01090-x 32264943 PMC7137345

[11] Pradies G. et al., “Clinical evaluation comparing the fit of all-ceramic crowns obtained from silicone and digital intraoral impressions based on wavefront sampling technology,” J. Dent. 43(2), 201–208 (2015). 10.1016/j.jdent.2014.12.007 25527248

[12] Jäger K. Vögtlin C., “Digital workflow with the Lava Chairside Oral Scanner C.O.S. and Lava technique,” Schweiz Monatsschr Zahnmed 122(4), 307–324 (2012). 22513752

[13] Mormann W. H., “The evolution of the CEREC system,” J. Am. Dent. Assoc. 137(suppl), 7S–13S (2006). 10.14219/jada.archive.2006.0398 16950932

[14] Zimmermann M. et al., “Intraoral scanning systems: a current overview,” Int. J. Comput. Dent. 18(2), 101–129 (2015). 26110925

[15] Mangano F. et al., “Intraoral scanners in dentistry: a review of the current literature,” BMC Oral Health 17(1), 149 (2017). 10.1186/s12903-017-0442-x 29233132 PMC5727697

[16] Dolcini G. A. Colombo M. Mangano C., “From guided surgery to final prosthesis with a fully digital procedure: a prospective clinical study on 15 partially edentulous patients,” Int. J. Dent. 2016, 7358423 (2016). 10.1155/2016/7358423 27493665 PMC4963589

[17] Nedelcu R. et al., “Accuracy and precision of 3 intraoral scanners and accuracy of conventional impressions: a novel *in vivo* analysis method,” J. Dent. 69, 110–118 (2018). 10.1016/j.jdent.2017.12.006 29246490

[18] Ahlholm P. et al., “Digital versus conventional impressions in fixed prosthodontics: a review,” J. Prosthodont. 27(1), 35–41 (2018). 10.1111/jopr.12527 27483210

[19] Imburgia M. et al., “Accuracy of four intraoral scanners in oral implantology: a comparative *in vitro* study,” BMC Oral Health 17(1), 92 (2017). 10.1186/s12903-017-0383-4 28577366 PMC5455075

[20] Roig E. et al., “*In vitro* comparison of the accuracy of four intraoral scanners and three conventional impression methods for two neighboring implants,” PLoS One 15(2), e0228266 (2020). POLNCL 1932-6203 10.1371/journal.pone.0228266 32106275 PMC7046187

[21] Sacher M. et al., “Comparing the accuracy of intraoral scanners, using advanced micro computed tomography,” Proc. SPIE 11113, 111131Q (2019). PSISDG 0277-786X 10.1117/12.2530728

[22] Rzanny A. et al., “PEEK: Werkstoffkundliche Eigenschaften – mit Blick auf die dentale Anwendung,” ZTM 2, 102–110 (2017).

[23] Althaus J. et al., “Micro- and nanostructured polymer substrates for biomedical applications,” Proc. SPIE 8339, 83390Q (2012). PSISDG 0277-786X 10.1117/12.915235

[24] Rebong R. E. et al., “Accuracy of three-dimensional dental resin models created by fused deposition modeling, stereolithography, and Polyjet prototype technologies: a comparative study,” Angle Orthod. 88(3), 363–369 (2018). 10.2319/071117-460.1 29509023 PMC8288316

[25] Flugge T. V. et al., “Precision of dental implant digitization using intraoral scanners,” Int. J. Prosthodont. 29(3), 277–283 (2016). 10.11607/ijp.4417 27148990

[26] Brogle-Kim Y.-C. et al., “Evaluation of oral scanning in comparison to impression using three-dimensional registration,” Proc. SPIE 8506, 85061R (2012). 10.1117/12.929727

[27] Castellini P. Scalise L. Tomasini E. P., “Teeth mobility measurement: a laser vibrometry approach,” J. Clin. Laser Med. Surg. 16(5), 269–272 (1998). JCLSEO 10.1089/clm.1998.16.269 9893508

